# The Roles of Histamine Receptor 1 (hrh1) in Neurotransmitter System Regulation, Behavior, and Neurogenesis in Zebrafish

**DOI:** 10.1007/s12035-023-03447-z

**Published:** 2023-07-20

**Authors:** Yuxiao Yao, Diego Baronio, Yu-Chia Chen, Congyu Jin, Pertti Panula

**Affiliations:** Department of Anatomy, University of Helsinki, POB 63, 00014 Helsinki, Finland

**Keywords:** Habenula, Hypocretin, Dopamine, Tyrosine hydroxylase, Anxiety

## Abstract

Histamine receptors mediate important physiological processes and take part in the pathophysiology of different brain disorders. Histamine receptor 1 (HRH1) is involved in the development of neurotransmitter systems, and its role in neurogenesis has been proposed. Altered HRH1 binding and expression have been detected in the brains of patients with schizophrenia, depression, and autism. Our goal was to assess the role of hrh1 in zebrafish development and neurotransmitter system regulation through the characterization of *hrh1*^−/−^ fish generated by the CRISPR/Cas9 system. Quantitative PCR, in situ hybridization, and immunocytochemistry were used to study neurotransmitter systems and genes essential for brain development. Additionally, we wanted to reveal the role of this histamine receptor in larval and adult fish behavior using several quantitative behavioral methods including locomotion, thigmotaxis, dark flash and startle response, novel tank diving, and shoaling behavior. *Hrh1*^−/−^ larvae displayed normal behavior in comparison with *hrh1*^+/+^ siblings. Interestingly, a transient abnormal expression of important neurodevelopmental markers was evident in these larvae, as well as a reduction in the number of tyrosine hydroxylase 1 (Th1)–positive cells, *th1* mRNA, and *hypocretin* (*hcrt*)-positive cells. These abnormalities were not detected in adulthood. In summary, we verified that zebrafish lacking *hrh1* present deficits in the dopaminergic and hypocretin systems during early development, but those are compensated by the time fish reach adulthood. However, impaired sociability and anxious-like behavior, along with downregulation of *choline O-acetyltransferase a* and *LIM homeodomain transcription factor Islet1*, were displayed by adult fish.

**Supplementary Information:**

The online version contains supplementary material available at 10.1007/s12035-023-03447-z.

## Introduction

Histamine exerts its biological effects through four G protein–coupled histamine receptors: HRH1, HRH2, HRH3, and HRH4 [1]. Previous studies have shown abnormal receptor radioligand binding and expression of HRH1 in different brain disorders, such as schizophrenia, depression, and autism [2]. A role for HRH1 in neurogenesis has also been established [3]. In vitro studies have shown that Hrh1 is expressed in neural stem cell niches in rodent brain and modulates neuronal differentiation [3]. Under traumatic brain injury or diabetic conditions, neurogenesis is promoted through HRH1 [4, 5]. *Hrh1*^−/−^mice display a reduced number of proliferating cells in the hippocampal dentate gyrus and a reduced number of newborn neurons but no changes in differentiation [6].

The development of distinct neurotransmitter systems is subject to HRH1 modulation. Patients with schizophrenia display reduced expression of HRH1 in cholinergic neurons as well as reduced expression of choline acetyltransferase (ChAT) [7]. Deletion of Hrh1 in the cholinergic neurons of mice leads to reduced acetylcholine content, ChAT expression in the basal forebrain, and reduced acetylcholine release in the medial prefrontal cortex [7]. In zebrafish, treatment with hrh1 antagonist pyrilamine reduces the number of these neurons [8], suggesting a role for HRH1 in the development of hypocretin neurons. This is supported by an early study indicating that total hypocretin content in the brains of *Hrh1*^−/−^ mice was reduced in comparison with WT animals [9]. Administration of HRH1 antagonist chlorpheniramine to pregnant rats reduces TH immunoreactivity in the striatum of 21-day-old pups. Additionally, striatal dopamine content, evoked [^3^H]-dopamine release and methamphetamine-stimulated motor activity were significantly lower in this offspring, indicating impaired dopaminergic transmission [10]. Interestingly, *Hrh1*^−/−^ mice present normal TH immunoreactivity in the striatum [11].*hrh1* mRNA expression in zebrafish can be detected in all developmental stages as early as 3 h post fertilization (hpf) [12]. *hrh1* is expressed prominently in the zebrafish habenula, anterior diencephalon, and locus coeruleus of the brain and other organs, including the liver, intestine, and spleen [12]. Few studies have assessed the role of hrh1 in zebrafish development and behavior using known receptor antagonists [8, 12–14]. However, interpreting the data generated by pharmacological treatments can be challenging as drugs can also have off-target effects and lack the specificity of genetic perturbations. Only one study has assessed the consequences of hrh1 loss of function through genetic manipulation. *hrh1*^−/−^ zebrafish generated by ENU mutagenesis show largely normal sleep/wake cycles and behavior, but the effects on brain development and adulthood remain unknown [15].

Our goal here was to investigate the role of hrh1 in zebrafish brain development and neurotransmitter system regulation through the characterization of *hrh1*^−/−^ fish generated by the CRISPR/Cas9 system. It was also relevant to find out if the lack of this histamine receptor, prominently expressed also in the human brain, will influence complex behaviors of adult fish.

## Results

### Eleven Nucleotide Deletion in hrh1 Mutant Zebrafish

**Fig. 1:**
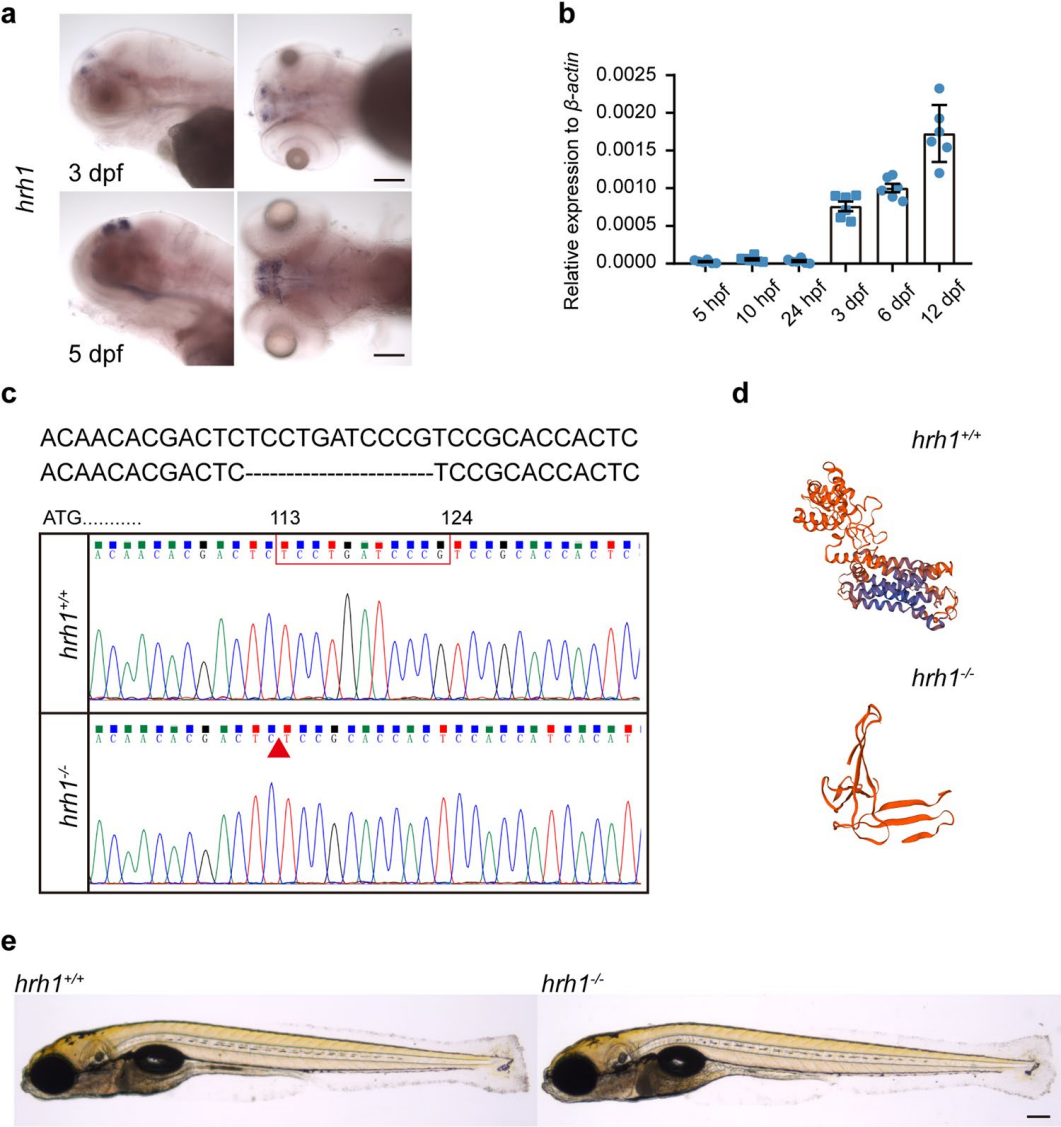
*hrh1* expression in Turku wt fishes and the mutation site of *hrh1*^−/−^ fish. **a** In situ hybridization of *hrh1* in Turku wt zebrafish at 3 dpf (lateral and dorsal view), 5 dpf (lateral and dorsal view). Scale bars: 50 µm. **b** qRT-PCR results of *hrh1* mRNA expression at 5 hpf, 10 hpf, 24 hpf, 3 dpf, 6 dpf, and 12 dpf. *N* = 6. Mean ± SEM. **c** The mutation site of *hrh1*^−*/*−^ zebrafish and genomic DNA sequencing results. **d** Predicated 3D modeling of *hrh1*^+*/*+^ and *hrh1*^−*/*−^ protein by using SWISS-model online. **e** Brightfield image of larval zebrafish at 6 dpf. Scale bar: 200 µm.

Zebrafish histamine receptor 1 (*hrh1*), cloned in 2007, is expressed from 3 hpf to adult stages [12]. Here, by using quantitative polymerase chain reaction (qPCR), it was verified that from 5 hpf to 12 dpf, *hrh1* mRNA expression increases, with the most significant increase taking place between 1 and 3 dpf (Fig. 1b). In situ hybridization showed that from 5 to 24 hpf, *hrh1* mRNA was already expressed at low level but widely in cells (Supplementary Fig. 1a). From 3 to 5 dpf, *hrh1* mRNA was strongly expressed in the brain (Fig. 1a). This expression continued to adulthood (Supplementary Fig. 1c). Analysis of dissected brains at 5 dpf and 12 dpf larval stages revealed that *hrh1* mRNA is expressed highly in the dorsal telencephalon, habenula, dorsal optic tectum, ventral telencephalon, diencephalic and thalamic regions, and lateral hypothalamus (Supplementary Fig. 1b).

To better understand the role of hrh1 in brain development and behavior, we used the CRISPR/Cas9 method to generate a *hrh1*^*−/−*^ zebrafish line. Sequencing showed an 11 base pair (bp) deletion from nucleotide 113 to 123 (NM_001042731.1) (Fig. 1c) in the mutant fish. The 11-bp nucleotide deletion resulted in a premature stop codon, which led to a truncated protein compared with the hrh1 protein structure as revealed by SWISS 3D modeling prediction (https://swissmodel.expasy.org/) (Fig. 1d). Gross morphology of the *hrh1*^*−*/*−*^ zebrafish larvae was not different from the wild-type siblings at 6 dpf (Fig. 1e). They grew normally, were fertile, and showed no obvious abnormal phenotype until adulthood.

As *hrh1* mRNA was expressed most prominently in the habenula, expression of the habenular markers potassium channel tetramerization domain containing 12.1 (*kctd12.1*) and G protein–coupled receptor 151 (*gpr151*) [16, 17] was first analyzed to reveal a potential role of hrh1 in development of the habenula. In situ hybridization and immunohistochemistry showed that both *kctd12.1* and *gpr151* expression in habenula at 6 dpf was identical in both genotypes (Supplementary Fig. 2a).

### Low Expression of Th1 and Few Hypocretin Cells in Larval *hrh1*^*−/−*^ Zebrafish

**Fig. 2:**
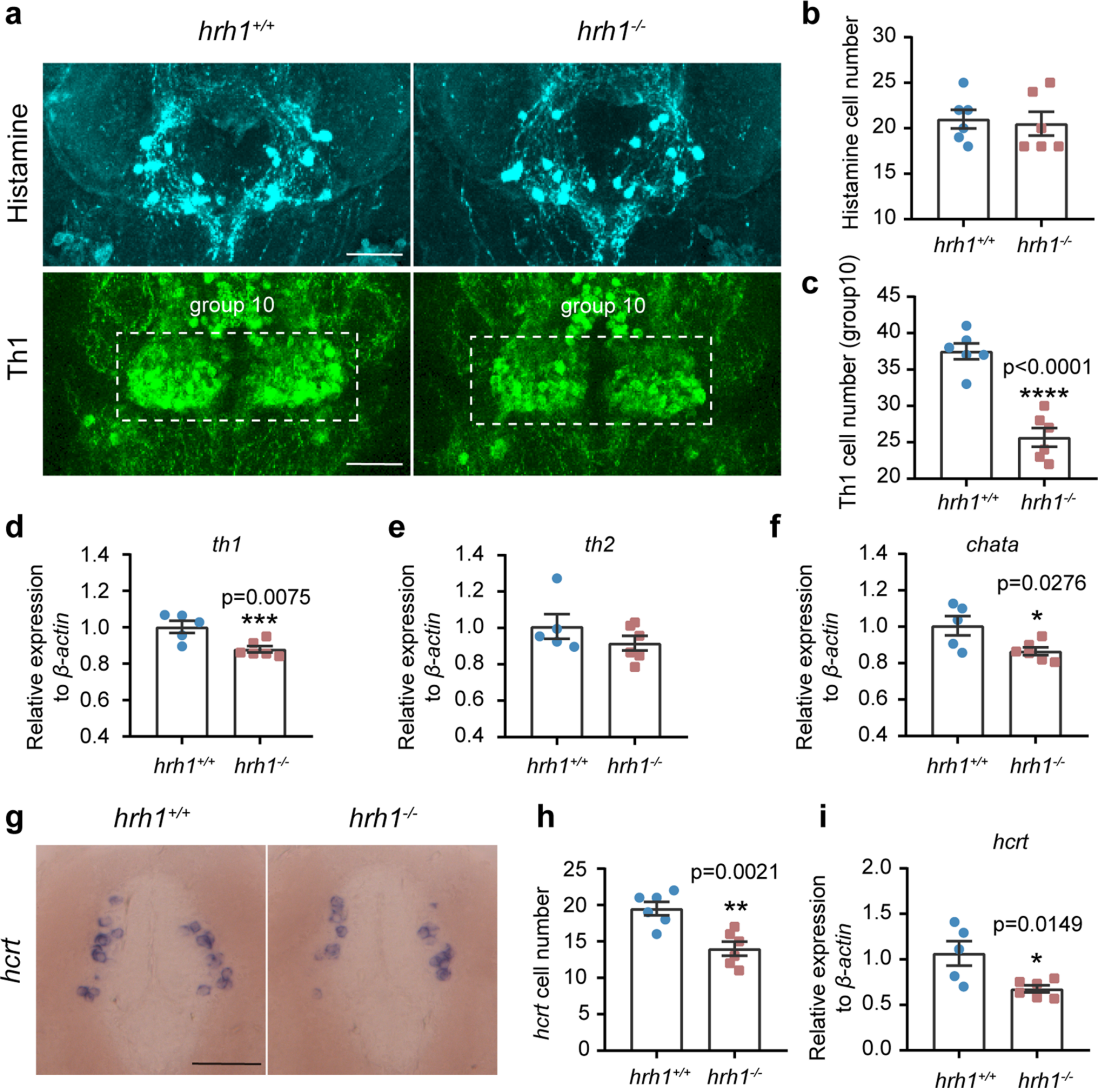
Histaminergic, dopaminergic, and hcrt systems in *hrh1*^−*/*−^ zebrafish. **a** Representative pictures (ventral view) for immunohistochemistry of histamine and Th1 in *hrh1*^+*/*+^ and *hrh1*^−*/*−^ 6 dpf zebrafish brains. Anterior to the left. Scale bars: 100 µm. **b** Histaminergic neuron numbers. *N* = 6. **c** Number of Th1-expressing neurons in group 10. *N* = 6. **d**–**f** qPCR results of *th1*, *th2*, *chata* in *hrh1*^+*/*+^ and *hrh1*^−*/*−^ in 6 dpf whole zebrafish. *N* (*hrh1*^+*/*+^) = 5, *N* (*hrh1*^−*/*−^) = 6. **g**–**h** Representative pictures (ventral view) for in situ hybridization of *hcrt* in *hrh1*^+*/*+^ and *hrh1*^−*/*−^ 6 dpf zebrafish brain and cell numbers. Anterior to the top. Scale bar 50 µm. *N* = 6. **i** qPCR results of *hcrt* in *hrh1*^+*/*+^ and *hrh1*^−*/*−^ in 6 dpf whole zebrafish. N (*hrh1*^+*/*+^) = 5, *N* (*hrh1*^−*/*^.^−^) = 6. All histograms are mean ± SEM, unpaired *t*-test, two-tailed. *0.01 < *P* ≤ 0.05; **0.001 < *P* ≤ 0.01; ***0.0001 < *P* ≤ 0.001; ****P ≤ 0.0001

Since neural circuits which use different transmitters to regulate complex behaviors, we found it relevant to study the role of hrh1 in regulation of some essential transmitter systems. As hrh1 is a receptor for histamine, the number and location of brain histamine neurons were examined in the *hrh1*^*−/−*^ zebrafish. At 6 dpf, identical anatomy of histamine-immunoreactive neurons in the posterior recess of the caudal hypothalamus was found in *hrh1*^*−/−*^ and *hrh1*^+*/*+^ larval brains (Fig. 2a). There were no differences in the number of histamine-ir cells between the *hrh1*^*−/−*^ and *hrh1*^+*/*+^ larvae at 6 dpf (Fig. 2b). In previous studies, we have reported that histaminergic neurons surround *tyrosine hydroxylase 1* (*th1*) expressing neurons in the posterior recess (*th1* group 10) [18]. In situ hybridization showed that the number of *th1* mRNA expressing neurons in group 10 [18] was significantly lower in *hrh1*^*−/−*^ than in *hrh1*^+*/*+^ larval brains (Fig. 2a, c) and at 6 dpf qPCR results also showed that *th1* mRNA level was significantly lower in whole *hrh1*^*−/−*^ larvae than in *hrh1*^+*/*+^ siblings (Fig. 2d). However, there was no difference in the adult brain (Supplementary Fig. 2b). No differences were found in expression levels of *th2* mRNA between the genotypes at larval or adult stage (Fig. 2e, Supplementary Fig. 2c).

Choline acetyltransferase (ChAT), the enzyme responsible for the biosynthesis of acetylcholine, is the most specific marker for monitoring cholinergic neurons in the central and peripheral nervous systems. qPCR results showed that *chata* mRNA level was significantly lower in *hrh1*^*−*/*−*^ larvae compared with *hrh1*^+*/*+^ larvae, and also in adult fish brain (Fig. 2f, Supplementary Fig. 2d). In situ hybridization of *hcrt* mRNA showed that the number of *hcrt* cells was about 35% higher in *hrh1*^+/+^ larval brains than in *hrh1*^*−*/*−*^ larvae (Fig. 2g, h). qPCR results showed that *hcrt* mRNA expression was indeed significantly lower in mutant larvae than in wild-type (wt) larvae (Fig. 2i), but the *hcrt* deficiency was restored in the adult brain (Supplementary Fig. 2e).

### Changes in* Nestin *and* notch1a *Expression in* hrh1*^*−/−*^Larval Brain

**Fig. 3:**
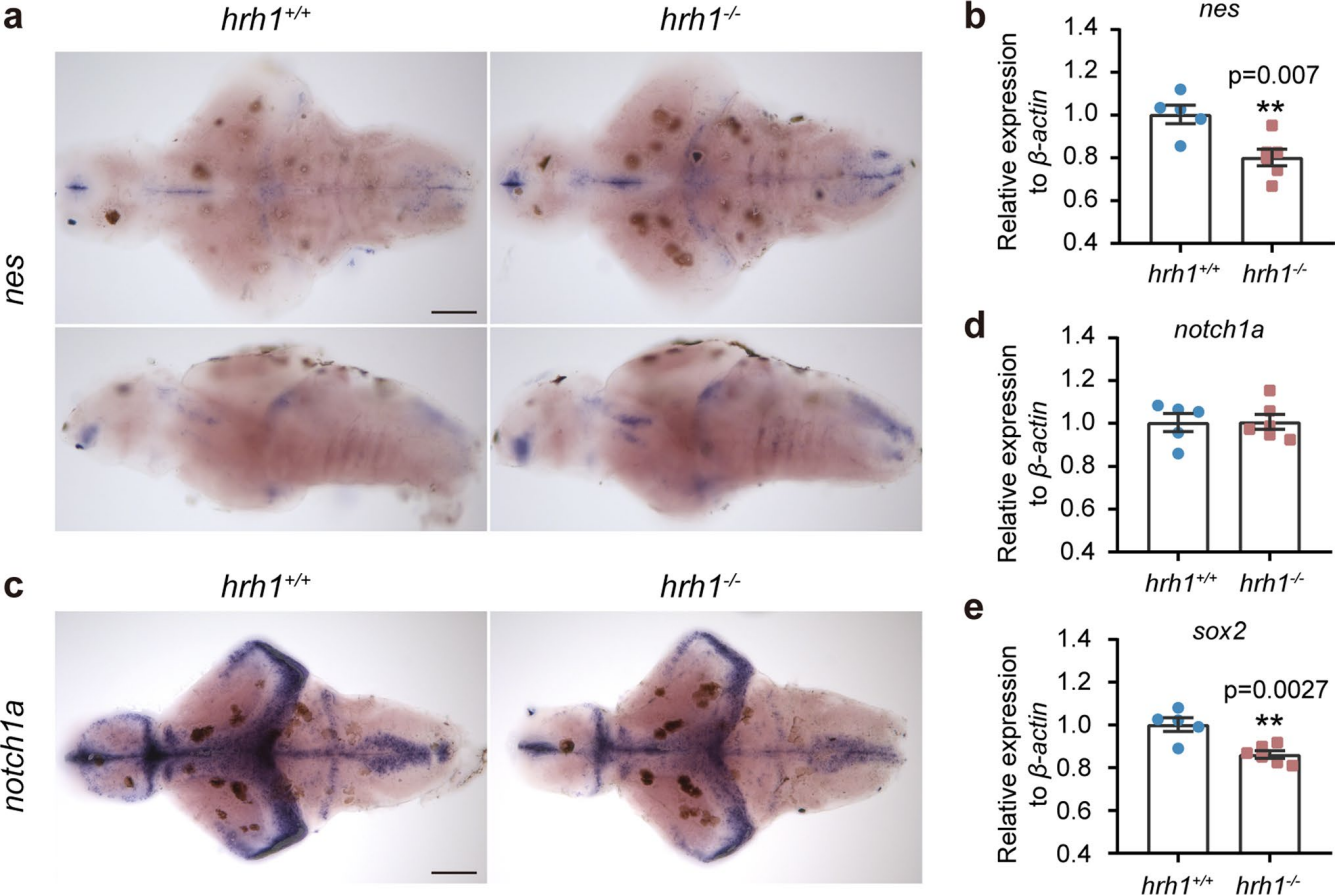
Neurogenesis markers in *hrh1*^−*/*−^ zebrafish. **a** Representative pictures for in situ hybridization of *nes* in *hrh1*^+*/*+^ and *hrh1*^−*/*−^ larval brain at 6 dpf in both dorsal and lateral view. Scale bar: 100 µm. Anterior to the left. *N* (*hrh1*^+*/*+^) = 5, *N* (*hrh1*^−*/*−^) = 6. **c** Representative pictures (dorsal view) for in situ hybridization of *notch1a* in *hrh1*^+*/*+^ and *hrh1*^−*/*−^ larval brain at 6 dpf. Anterior to the left. Scale bar: 100 µm. *N* (*hrh1*^+*/*+^) = 8, *N* (*hrh1*^−*/*−^) = 8. **b**, **d**, **e** qPCR results of *nes*, *notch1a*, *sox2* in whole larval zebrafish at 6 dpf. *N* (*hrh1*^+*/*+^) = 5, *N* (*hrh1*^−*/*^.^−^) = 6. All bar graphs show mean ± SEM, unpaired *t*-test, two-tailed. **0.001 < *P* ≤ 0.01

Based on evidence that histamine has a role in neurogenesis and on the literature indicating that HRH1 is an important modulator in this process [3], we aimed to verify the effects of *hrh1* deletion on zebrafish brain development. The stem cell marker *nestin* (*nes*) expression was higher in *hrh1*^*−*/*−*^ larval brain compared with *hrh1*^+*/*+^ larvae (Fig. 3a). However, in whole 6 dpf, larval fish *nes* mRNA expression was lower in mutants than in *hrh1*^+*/*+^ larvae (Fig. 3b). No significant difference in expression was found in adult zebrafish brain between the genotypes (Supplementary Fig. 3a). Considering that *nes* is also expressed strongly in cranial ganglia, the gut, and craniofacial mesenchyme outside the brain [19], possible abnormal expression in the periphery might explain the contradictory results obtained by in situ hybridization and qPCR.

The expression of early neuronal marker *notch1a* was weaker in the brains of *hrh1*^*−*/*−*^ larvae when compared with their *hrh1*^+/+^ siblings (Fig. 3c). Notch1 signaling contributes to the maintenance of neural progenitor cells in the undifferentiated state [20]. In addition to this well-established role for notch1, it is also involved in cell proliferation and apoptotic events [21]. However, when we utilized pools of whole larvae as samples for qPCR analysis, we did not detect a difference in the amount of *notch1a* transcript between genotypes (Fig. 3d). Similarly, as *nes*, it is worth mentioning that *notch1a* mRNA is abundant in different larval tissues and the qPCR may be unable to reflect a possible difference in the brains of mutants and wt siblings, considering that whole organisms were used as samples [22, 23]. Interestingly, when we utilized adult zebrafish brains as samples, there was no difference between genotypes suggesting a transient difference during early stages (Fig. 3d, Supplementary Fig. 3b).

Another stem cell marker *sox2* showed significantly lower expression in whole *hrh1*^*−*/*−*^ larvae than in *hrh1*^+/+^ larvae, but no differences were seen in adult zebrafish brains (Fig. 3e, Supplementary Fig. 3c). Altogether, our data indicate that the abnormalities in developmental markers in zebrafish caused by *hrh1* deletion are limited to an early stage of development and are likely to be compensated by a mechanism that remains to be determined.

### Proliferation and Differentiation in hrh1^−/−^ Zebrafish Larval Brain

**Fig. 4:**
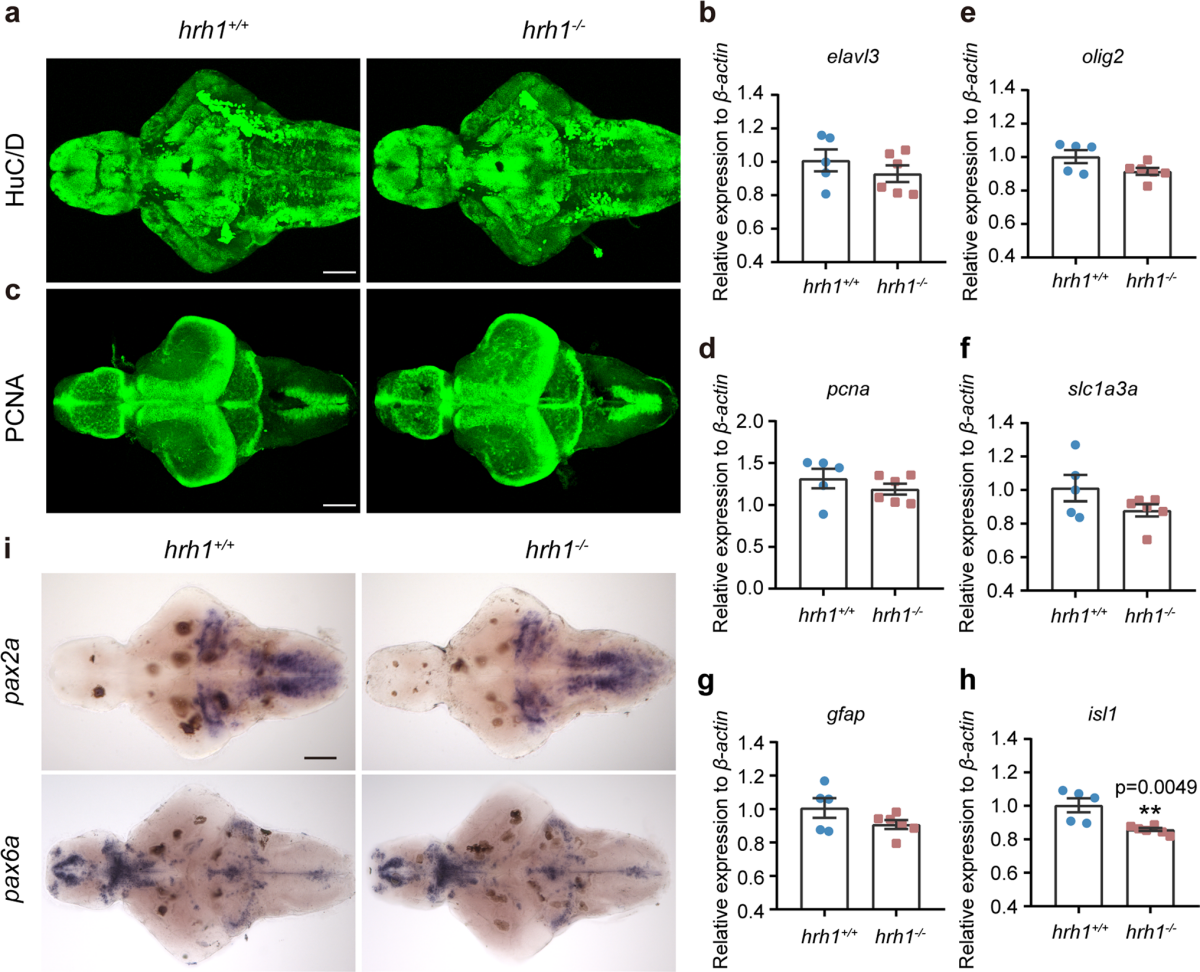
Proliferation and differentiation markers in *hrh1*^−/−^ zebrafish. **a** Representative pictures (ventral view) for immunohistochemistry of HuC/D in *hrh1*^+*/*+^ and *hrh1*^−*/*−^ larval brain at 6 dpf. Anterior to the left. Scale bar: 100 µm. *N* = 6. **c** Representative pictures (dorsal view) for immunohistochemistry of PCNA in *hrh1*^+*/*+^ and *hrh1*^−*/*−^ larval brain at 6 dpf. Anterior to the left. Scale bar: 100 µm. *N* = 6. **i** Representative pictures (ventral view) for in situ hybridization of *pax2a* and *pax6a* in *hrh1*^+*/*+^ and *hrh1*^−*/*−^ larval brain at 6 dpf. Anterior to the left. Scale bar: 100 µm. **b**, **d**–**h** qPCR results of *elavl3*, *pcna*, *olig2*, *slc1a3a*, *gfap*, and *isl1* in whole larval zebrafish at 6 dpf. *N* (*hrh1*^+*/*+^) = 5, *N* (*hrh1*^−*/*^.^−^) = 6. All histograms are exhibit as mean ± SEM, unpaired *t* test, two tailed. **0.001 < *P* ≤ 0.01

Since hrh1 is known to regulate neuronal proliferation and differentiation in mammalian brain, we used the *hrh1*^*−*/*−*^ zebrafish to study essential genes in neuronal and glial differentiation. As *notch1a* signaling downregulation could indicate an increase in differentiation, the HuC/D antibody was used to study the maturity of neurons in larval brains. The results showed a similar distribution of HuC/D expression in *hrh1*^*−/−*^ and *hrh1*^+*/*+^ larval brains (Fig. 4a). No differences were found in the HuC/D (*elavl3*) mRNA expression in whole larvae or adult brains (Fig. 4b, Supplementary Fig. 4a) either. Furthermore, the proliferation marker PCNA was similarly expressed at both protein, and mRNA levels in larval and adult brains (Fig. 4c, d, Supplementary Fig. 4b). Other cellular markers expressed in glial cells *slc1a3a* (predicted to enable l-glutamate transmembrane transporter activity and present in glioblasts), oligodendrocyte marker *olig2*, and astrocyte marker *gfap* showed no differences between the genotypes in whole larvae or adult brains (Fig. 4e–g, Supplementary Fig. 4 d-f). Developmental neuronal markers *pax2a* and *pax6a* (Fig. 4i) were also similarly expressed in the genotypes. Surprisingly, we found that expression of motor neuron marker *isl1* was significantly lower in both whole larval and adult brains in *hrh1*^*−*/*−*^ than in *hrh1*^+*/*+^ fish (Fig. 4h, Supplementary Fig. 4c).

### hrh1^−/−^ Zebrafish Larvae Showed No Significant Behavioral Phenotype

**Fig. 5:**
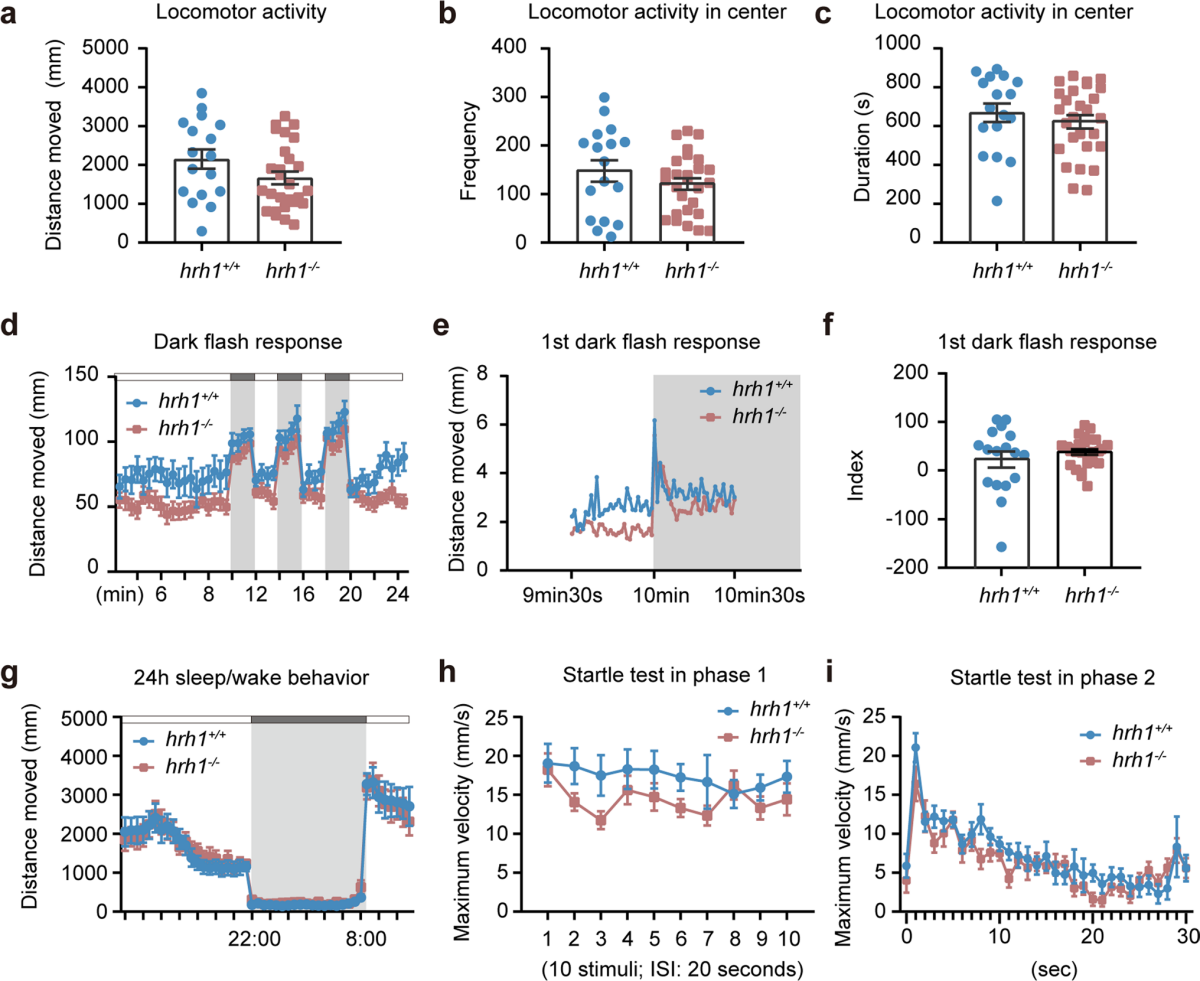
Larval behavior of *hrh1*^+*/*+^ and *hrh1*^−*/*−^ fish. **a** Total distances moved of *hrh1*^+*/*+^ and *hrh1*^−*/*−^ larvae at 6 dpf in an open field for 15 min. **b** The frequency of zebrafish entering the center zone of an open field. **c** The duration of zebrafish staying in the center zone of open field. **d** Dark flash responses of *hrh1*^+*/*+^ and *hrh1*^−*/*−^ zebrafish. Each dark period lasted 2 min. Distance moved during every 30-s period is indicated on the *Y* axis. **e** Distance moved/s of *hrh1*^+*/*+^ and *hrh1*^−*/*−^ in the first dark response period. **f** The index of the first dark response (distances moved in the dark (30 s) minus distances moved in the light (30 s)). **a**–**f** *N* (*hrh1*^+*/*+^) = 17, *N* (*hrh1*^−*/*−^) = 27. **g** Sleep-like behavior from 5 to 6 dpf of *hrh1*^+*/*+^ and *hrh1*^−*/*−^ zebrafish. Distance covered during every 30-min period is indicated on the *Y* axis. *N* (*hrh1*^+*/*+^) = 16, *N* (*hrh1*^−*/*−^) = 14. **h**–**i** Mean values for maximum velocity during 10 acoustic/vibrational stimuli with a 20-s interstimulus interval (ISI) (h) and 30 acoustic/vibrational stimuli with 1 s ISI. *N* (*hrh1*^+*/*+^) = 14, *N* (*hrh1*^−*/*−^) = 15. Mean ± SEM, two‐way ANOVA

Since differences in gene expression were found in *hrh1*^+*/*+^ and *hrh1*^*−*/*−*^ larval zebrafish brains, we reasoned that this might lead to abnormalities in behavior. Locomotor activity, measured as total distance moved, did not significantly differ between *hrh1*^+*/*+^ and *hrh1*^*−*/*−*^ larvae at 6 dpf (Fig. 5a). During the same trial, we also assessed thigmotaxis, a behavior in which animals tend to avoid the center of the arena and is generally associated with an anxious-like phenotype [24]. *hrh1*^*−*/*−*^ larvae and *hrh1*^+*/*+^ siblings did not statistically differ when the frequency and cumulative time spent in the center of the arena were measured (Fig. 5b, c).

To explore the reaction to light/dark transitions, larvae were subjected to 2-min light/dark periods. All larvae reacted with similar increases of movement (dark flash responses) to sudden darkness (Fig. 5d). Further analysis at 1-s resolution of the first dark period (from 9 min 30 s to 10 min 30 s refer to Fig. 5d) and first dark response also revealed no differences between the genotypes (Fig. 5e, f). Twenty-four-hour sleep/wake behavior under light conditions, which mimic the daily circadian changes in illumination, revealed no differences between the genotypes (Fig. 5g).

In the acoustic/vibrational startle test [25], larvae were maintained in the test box for 30-min-long habituation, then the system tapped the plates with the larvae in two phases. In the first phase, 10 repeated taps occurred at 20-s intervals. We observed that before the taps, no differences in the velocity were found between *hrh1*^+*/*+^ and *hrh1*^*−*/*−*^ larvae at 6 dpf, suggesting no differences in the basal functioning of the Mauthner cell-based startle circuitry. *hrh1*^*−*/*−*^ larvae seemed to habituate to repeated exposure to the acoustic/vibrational stimuli at 20-s inter-stimulus-interval (ISI), similar to *hrh1*^+/+^ siblings. In the second phase, 30 taps occurred at 1-s intervals, and *hrh1*^*−*/*−*^ larvae and *hrh1*^+*/*+^ siblings presented similar behavior (Fig. 5h, i).

### Hrh1^+/+^ and hrh1^−/−^ adult fish display differences in novel tank diving test and shoaling behavior

**Fig. 6:**
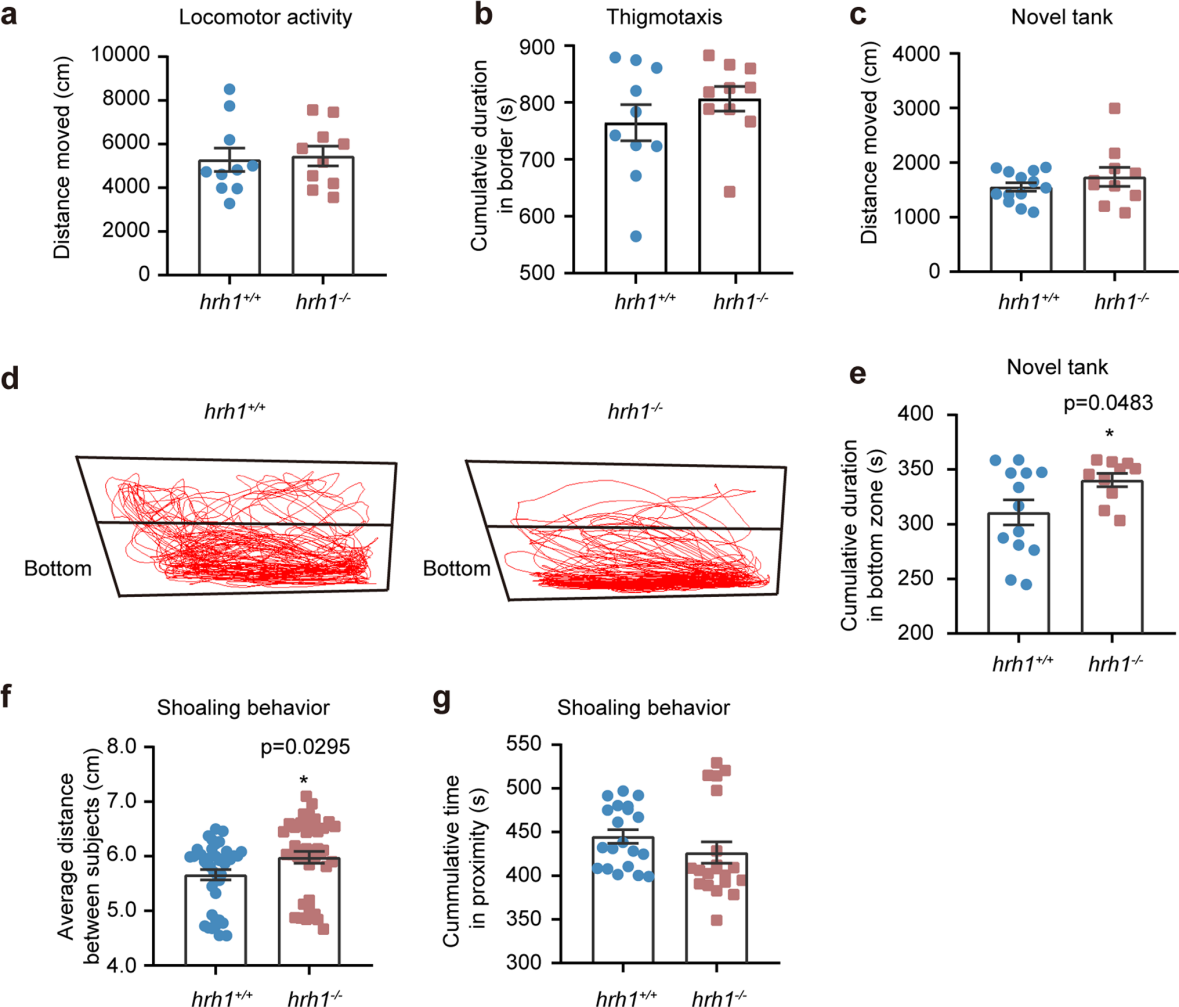
Adult behavior of *hrh1*^+*/*+^ and *hrh1*^−*/*−^ zebrafish. **a** Total distances moved in an open-field tank in 15 min in total by adult *hrh1*^+*/*+^ and *hrh1*^−*/*−^ fish. **b** Duration of *hrh1*^+*/*+^ and *hrh1*^−*/*−^ fish in the border zone. *N* = 10. **c** Total distances moved in the novel tank in 6 min in total. *N* (*hrh1*^+*/*+^) = 13, *N* (*hrh1*^−*/*−^) = 10. **d** Representative tracking map of novel tank for *hrh1*^+*/*+^ and *hrh1*^−*/*−^ adult zebrafish. **e** Statistics of cumulative durations in the bottom zone for *hrh1*^+*/*+^ and *hrh1*^−*/*−^ adult zebrafish. **f**–**g** Statistics of the distances between subjects and cumulative duration in proximity for *hrh1*^+*/*+^ and *hrh1*^−*/*−^ adult zebrafish. *N* = 20. Mean ± SEM, unpaired *t* test, two tailed. *0.01 < *P* ≤ 0.05; no stars mean no significant differences

Locomotor activity, measured as total distance moved, did not significantly differ between *hrh1*^+/+^ and *hrh1*^*−*/*−*^ adult fish during a 15-min trial (Fig. 6a). During the same trial, we also assessed thigmotaxis. Still, no difference between genotypes was detected when this behavior was analyzed. Both *hrh1*^+/+^ and *hrh1*^*−*/*−*^ adult fish stayed during most of the trial in the zone close to the arena wall (border) (Fig. 6b).

The novel tank was digitally divided into two zones, and representative swimming tracks of *hrh1*^+/+^ and *hrh1*^*−*/*−*^ adult fish during the 6-min trial are shown (Fig. 6d). Both genotypes displayed similar locomotor activity during the trial when *hrh1*^+/+^ and *hrh1*^*−*/*−*^ fish were compared (Fig. 6c). However, *hrh1*^*−*/*−*^ adult zebrafish stayed significantly longer time in the bottom of the tank when compared with *hrh1*^+/+^ siblings (Fig. 6e).

We also tested the shoaling behavior of adult *hrh1*^*−*/*−*^ and *hrh1*^+/+^ zebrafish. The results showed that the distances between subjects were significantly increased in the *hrh1*^*−*/*−*^ group (Fig. 6f) although the cumulative duration in proximity was not significantly different between the genotypes (Fig. 6g). These results indicate that this form of social interaction is abnormal in *hrh1*^*−*/*−*^ zebrafish.

## Discussion

In this study, we characterized morphological, behavioral, and neurological aspects of *hrh1*^*−*/*−*^ zebrafish in different stages of development. *hrh1*^*−*/*−*^ larvae displayed normal behavior in comparison with *hrh1*^+/+^ siblings. Interestingly, a transient downregulation of important neurodevelopmental markers was noticed in *hrh1*^*−*/*−*^ larvae, as well as a reduction in the number of Th1-positive cells, *th1* mRNA, and *hcrt*-positive cells. No abnormalities in this regard were detected in adulthood. However, impaired sociability and anxious-like behavior were displayed by adult fish.

Previously, we reported on *hrh1* expression from 3 hpf [12]. Here, using in situ hybridization, we validated that at 5 hpf, *hrh1* mRNA was already expressed in most cells, and the broad expression continued at least until 24 hpf. From 3 dpf, *hrh1* was expressed mainly in the brain. The dissected larval brain showed that *hrh1* is expressed in the dorsal and ventral telencephalon, diencephalic and thalamic regions, and lateral hypothalamus, as reported before, but also in the neuron progenitor pool of dorsal optic tectum [26, 27]. Thus, the expression pattern agrees with the potential role of hrh1 in zebrafish larval brain neurogenesis.

Histamine induces neural stem cell proliferation and neuronal differentiation by activating distinct receptors. HRH2 activation has a role in cell proliferation, and HRH1 activation seems critical for neuronal differentiation and cell survival [3]. In vitro and in vivo studies have been used to evaluate the role of HRH1 in cell differentiation. Activation of HRH1 of neural stem cells in culture significantly increases the number of differentiated neurons [28]. *Hrh1* KO mice showed a reduced number of BrdU-labeled cells in the hippocampus [6]. However, analysis of the double labeling of BrdU with either NeuN as a marker for mature neurons or GFAP as an astroglial marker revealed no difference between WT and *Hrh1* KO mice in the percentage of cells that developed into neurons or astrocytes. Our qPCR result shows that *sox2* is downregulated in *hrh1*^*−*/*−*^ larvae. Expression of *notch1a* was also weaker in the brains *hrh1*^*−*/*−*^ larvae than in *hrh1*^+/+^ siblings*.* One of the roles of sox2 and notch1 signaling is the maintenance of stem cells. In zebrafish, *sox2* knockdown and *notch1a* inhibition through treatment with DAPT lead to upregulation of neurogenesis [29]. We wanted to verify if the lower expression of these factors in the *hrh1*^*−*/*−*^ larvae would increase stem cell differentiation. However, the expression of the pan-neuronal marker HuC/D, GFAP, and *olig2* were similar when genotypes were compared. Additionally, proliferation seemed unaffected as PCNA expression was unchanged when *hrh1*^*−*/*−*^ larvae were compared with *hrh1*^+/+^ siblings. In mice, *sox2* deficiency impairs neuronal differentiation, and Notch1-deficient cells do not complete differentiation but are eliminated by apoptosis, resulting in a reduced number of neurons in the adult cerebellum [30, 31]. Interestingly, a reduced number of Th1-positive cells in the hypothalamic group 10 and *hypocretin*-positive cells, as well as a downregulation of the cholinergic neurons marker *chata*, were detected in the *hrh1*^*−*/*−*^ larvae. In situ hybridization results showed that the expression of *pax2a* and *pax6a*, other regulators in the neuronal fate determination, was similar in both genotypes.

The alterations in early developmental markers in the *hrh1*^*−*/*−*^ brains seemed mild and transient, as we did not detect major abnormalities in adult brains. It is noteworthy that only a few studies approach the role of histamine receptors on zebrafish brain development [8, 32, 33]. For instance, the pattern of expression of *hrh2* in the zebrafish is yet to be elucidated. Additionally, many genes are duplicated in the teleost, and the paralogs could have distinct functions. Thus, possible compensatory effects during development by the activation of other histamine receptors or G protein–coupled receptors with related functions in the brain of *hrh1*^*−*/*−*^ larvae should not be discarded.

Tyrosine hydroxylase (Th), the enzyme responsible for dopamine production, has two non-allelic forms (Th1 and Th2) in zebrafish [34, 35]. *Th1* and *th2* mRNAs show a complementary expression patterns, and *th1* expression is more widespread than *th2*, with more neuronal groups expressing the enzyme [35]. Here, we found that *hrh1*^*−*/*−*^ larval zebrafish showed a decreased expression of *th1* but not *th2*. The number of *th1* positive neurons was also significantly smaller in the zebrafish hypothalamus in cell group 10 of the posterior hypothalamus in *hrh1*^*−*/*−*^ larvae than in *hrh1*^+/+^ larvae. It has been previously reported that administration of HRH1 antagonist chlorpheniramine to pregnant rats leads to a significant reduction in TH immunoreactivity in the substantia nigra *pars compacta* and dorsal striatum of 21-day-old pups [10]. Additionally, striatal dopamine content evoked [^3^H]-dopamine release and methamphetamine-stimulated motor activity was significantly lower in this offspring, indicating impaired dopaminergic transmission. However, when TH immunoreactivity was evaluated in the striatum of adult *Hrh*1 KO mice, no statistically significant differences were detected in comparison with WT animals [11] but an increase of TH immunoreactivity was detected in the basolateral anterior, basolateral ventral, and cortical amygdaloid nuclei [36].

The acetylcholine, dopamine, histamine, and hypocretin systems are all long-ranged, highly connected projections to diverse brain regions regulating arousal and memory [37, 38]. It is known that in rodents histamine stimulates strongly septal cholinergic neurons through histamine receptor Hrh1 [39], and *hrh1* mRNA is expressed in these large septal neurons [40]. Hrh1 deletion in cholinergic mouse neurons leads to lower levels of acetylcholine in the basal forebrain (BF) and medial prefrontal cortex [7]. Additionally, in mutant mice, protein expression levels of ChAT were downregulated in the BF and prefrontal cortex [7]. Here, we found that choline acetyltransferase a (*chata*) mRNA expression was lower in whole *hrh1*^*−*/*−*^ larvae and adult zebrafish brains than in *hrh1*^+/+^ fish. Interestingly, larval and adult *hrh1*^*−*/*−*^ fish presented *isl1* downregulation. Conditional deletion of *isl1* in the brain leads to a loss of cholinergic interneurons in the striatum and a loss of cholinergic projection neurons in the nucleus basalis [41]. Furthermore, isl1 is involved in multiple aspects of motor neuron development [42]. However, *hrh1*^*−*/*−*^ larval and adult basic locomotor activity and response to acoustic/vibrational stimuli, which is mediated by Mauthner cell that forms excitatory synapses on primary motor neurons [43], are not statistically different from the *hrh1*^+/+^ siblings.

In zebrafish, the hypocretin system sends projections to aminergic nuclei, raphe, locus coeruleus, the mesopontine-like area, dopaminergic clusters, and histaminergic neurons [38]. The hypocretin and histamine systems drive wakefulness [44, 45]. Here, we found that hypocretin cells and mRNA expression are significantly decreased in *hrh1*^*−*/*−*^ larvae compared with *hrh1*^+*/*+^ larvae. These results agree with the lower brain hypocretin content in the brains of *hrh1*^*−/−*^ mice than in *hrh1*^+*/*+^ mice, and with our previous data that treatment with hrh1 antagonist pyrilamine decreases the number of hypocretin cells in developing zebrafish brain [8, 46]. However, the 24-h track of locomotor activity, the dark flash response, and tapping responses of *hrh1*^*−/−*^ zebrafish larvae did not differ from that of *hrh1*^+/+^ larvae. Since *hrh1*^*−/−*^ adult zebrafish presented restored levels of *hcrt* mRNA transcript relative to *hrh1*^+/+^ siblings, the role of HRH1 in hcrt regulation appears to be transient and limited to early developmental stages.

Motivated by the increasing of evidence pointing to a possible role of the histaminergic system in the pathophysiology of autism spectrum disorder (ASD) [47, 48] and disorders that share similar symptomatology, including schizophrenia [49]. In attention-deficit hyperactivity disorder [50] and Gilles de la Tourette syndrome [51], we decided to investigate the consequences of *hrh1*-loss of function on relevant behaviors. Interestingly, when sociability was assessed through the shoaling test, adult *hrh1*^−/−^ zebrafish displayed looser shoals compared with cohorts formed by *hrh1*^+/+^ siblings, indicating impaired social behavior. Although a specific role for HRH1 in social interaction has not been established, it is becoming evident that histaminergic neurotransmission contributes to this behavior. We have reported deficits in the social behavior and histaminergic system of zebrafish embryonically exposed to valproic acid, an ASD-related drug [52]. The antagonism of Hrh3 has recently received attention in preclinical studies as a potential therapeutical approach to treat ASD-like symptoms [53, 54]. Low HRH1 binding was observed in the frontal and prefrontal cortices and cingulate cortex in the brains of people with schizophrenia, a disorder in which patients often present social withdrawal and impaired communication [49]. Recently, it has been shown that patients with schizophrenia having negative symptoms present decreased HRH1 expression in BF cholinergic neurons and that deletion of Hrh1 in mice cholinergic neurons reduces their interest in social novelty [7]. Furthermore, in zebrafish, hrh1 is prominently expressed in the habenula, a structure directly linked with processing social information [55].

Here we also report an anxious-like phenotype and decreased exploratory activity of *hrh1*^−/−^ adult zebrafish during a novel tank diving test compared with their *hrh1*^+/+^ siblings. Inoue et al. reported that Hrh1-deficient mice had decreased ambulation and time of rearing during a 30-min trial in a new environment [56], indicating that through Hrh1, the histaminergic neuron system plays an important role in locomotor activities related to emotion. Additionally, during a passive-avoidance test, those mice showed more frequent urination and defecation and decreased movements compared with wt mice [56]. After 4 trials with a 24-h intertrial interval, Zlomuzica et al. reported that *Hrh1* KO and WT mice showed similar behavioral habituation to the open‐field. However, in the same study, *hrh1*-KO showed impaired spatial novelty‐induced behavior in the Y‐maze [36]. When *Hrh1* KO and WT mice were tested in the light/dark test, *Hrh1* KO mice did not show increased/or decreased emotionality in terms of brightly lit spaces [57]. The initial preference of the adult zebrafish for the bottom of a novel tank, and subsequent exploration of the rest of the tank, is commonly interpreted as a precautionary antipredator response followed by alleviation of anxiety, respectively [58]. In the case of *hrh1* mutants, it could reflect a lack of motivation toward novelty exploration. The fact that in our study *hrh1*^+/+^ and *hrh1*^−/−^ zebrafish did not show a difference in locomotor activity during the open-field test could be explained by the period of adaptation that both genotypes had in the arena prior to the test, differently than the novel tank test where fish are immediately tracked after being placed in a new environment.

We reported for the first time significant differences in key neurotransmitter systems and neurogenesis markers of *hrh1*^−/−^ zebrafish during the development that were compensated by the time the fish reached maturity. Nevertheless, adult animals displayed behavioral alterations regarding social interaction and exploratory activity. Further in-depth studies are necessary to determine to which extent hrh1 deficiency and the early developmental abnormalities are responsible for these phenotypes.

## Methods

### Zebrafish Maintenance and Gene-Modified Fish

Larval and adult zebrafish were raised under standard laboratory conditions at 28 ℃. Turku wild-type fishes have been maintained in the lab for more than 20 years. All animal care and experimental procedures complied with the ethical guidelines of the European convention, and a permit was obtained for the experiments from the Regional District of Southern Finland (ESAVI/13090/2018). *hrh1* mutation was introduced by CRISPR/Cas9. The target site was chosen based on the website (http://chopchop.cbu.uib.no/). Target sequence was 5′-3′ GATCCCGTCCGCACCACT. In vitro transcriptions of Cas 9 and gRNA preparation were as previously described [59]. Microinjection into zebrafish embryos at the single-cell stage was performed with 400 μg/mL Cas9 mRNA and 70 μg/mL sgRNA targeting hrh1. Genotyping of the founder fish, F1, F2, and F3 generation was done as before [60] To describe briefly, DNA was extracted from individual injected eggs and non-injected controls at 24 hpf. Hrh1 high-resolution melting (HRM) primers (F: CAGTCACACCTTGTCTTTGGTC; R: CCAAATTGAGCGGCAT CACG) cover the gRNA target site used for PCR amplification to be analyzed via Sanger sequencing and HRM analysis. Mutations were recognized as multiple sequencing peaks at the sgRNA target site. When the mutation efficiency was over 50%, the remaining injected eggs were raised to adulthood and outcrossed with wild-type fish to generate F1 progeny. F1 larval tails were cut at 3 dpf after anesthetized with 0.02% Tricaine. Then the tail biopsies were collected to extract DNA. All the cut larvae were placed in an individual well of a 24-well plate with the fresh embryonic medium until 5 dpf. F1 genotyping was done by real-time PCR-high-resolution melt (RT-PCR-HRM) assays and DNA sequencing with HRM primers. Each target locus was PCR amplified from individual genomic DNA with HRM primers to identify mutated alleles from single embryos. PCR products were then cloned and sequenced. Mutated alleles were identified by comparison with the wild-type sequence. The identified F1, which carry the same mutations, were pooled together, raised to adulthood, and then outcrossed with wild-type fish again to get the F2 generation. Like the F1 generation, F2 larvae were cut and analyzed by HRM. Identified heterozygous F2 siblings were put together in a large tank and raised to adulthood. Crossing the F2 heterozygous females and males, the F3 generation larvae produced wild-type, heterozygous, and homozygous offspring. Again, the larval tails were cut and analyzed by HRM. Wild-type and homozygous fish were used for experiments at 6 dpf or raised to adulthood to do adult behavior.

### In Situ Hybridization

Whole larval fish from 5 hpf to 12 dpf were fixed in 4% paraformaldehyde (PFA) overnight at 4℃. On the second day, the PFA was changed to PBS and the samples were washed 3 times. Then larval brains were dissected and washed with PBS 3 times. Whole embryos or dissected brains were hybridized following the Thisse and Thisse protocol [61] except for prehybridization and hybridization at 60℃ instead of 70℃. All the probes and cloning primers are listed in Table 1. The PCR products were cloned into a pGEM-Teasy vector and sequenced for synthesizing the probes for in situ hybridization. Zebrafish *hrh1*, *hcrt*, *notch1a*, *pax2a*, and *pax6a* probes were those used and published before [8, 62–64].

**Table 1:** Primers for probe cloning

	Forward primer	Reverse primer
*nes* probe	GTACCAGATGCTAGAGCTGAACCACCGCCTT	GCATCTGCCTCTTGATCCTCGTGCTCTCCAG
*gpr151* probe	GGAGAGCAGTGGATACCAGC	GAGTGAGCCTCCGCCATAAA
*kctd12.1* probe	GTGGAATATCAAACGGCGCT	TCAAACGCCTGTTCGAGGAA

### Quantitative Real-Time PCR

Total RNA was extracted from 5 pooled larval fish or 1 dissected adult brain using the RNeasy mini Kit (Qiagen). cDNA was synthesized by using SuperScriptTM III reverse transcriptase (Invitrogen). Real-time PCR was conducted in a LightCycler® 480 instrument (Roche, Mannheim, Germany) using the Lightcycler®480 SYBR Green I master mix (Roche, Mannheim, Germany) according to the manufacturer’s instructions. mRNA levels were normalized to an average of *β-actin* as an internal control. The primers used are shown in Table 2. The qPCR analysis was done using 2 − ΔΔCT method as Livak and Schmittgen reported before [65]. Briefly, the wt fish was normalized to a comparative average of 1 and values of hrh1^−/−^ fish were correlated to those of hrh1^+/+^ fish.

**Table 2:** QPCR primers

*β-actin*	CGAGCAGGAGATGGGAACC	CAACGGAAACGCTCATTGC
*hrh1*	TGCCAACAGCCAGTTCAAGA	ATCCCCAGGAAAATGCCCAG
*th1*	GACGGAAGATGATCGGAGACA	CCGCCATGTTCCGATTTCT
*th2*	CTCCAGAAGAGAATGCCACATG	ACGTTCACTCTCCAGCTGAGTG
*chata*	TGCGAGAGATTGCCAAGGAG	CTCAGTGGTAGGAACCTGGC
*hcrt*	AGAAACGACTCTTCCGTCGC	CGGCTTGATTCCGTGAGTTG
*nes*	GAGAGCACGAGGATCAAGAGG	GGGTGTTTACTTGGGCCTGAAGAT
*notch1a*	AGAGCCGGATTCAGCGGTC	TTACAGGGACGTGGAGAACAAG
*sox2*	CCTATTCGCAGCAAAGCACG	GGAATGAGACGACGACGTGA
*elavl3*	AGACCCAGCGCTTCAGATTC	TAGACGAAGATGCACCAGCC
*pcna*	ACGCCTTGGCACTGGTCTT	CTCTGGAATGCCAAGCTGCT
*olig2*	AAGAGGTGCAGATATGCGGG	TCTCGCGGCTGTTGATCTTT
*gfap*	GAAGCAGGAGGCCAATGACTATC	GGACTCATTAGACCCACGGAGAG
*slc1a3a*	ATCGCCGTCTTTATCGGCAT	GAGATCGAGGAAGGCGTCTG
*isl1*	CATCCGGCGAGACCTTTACA	GGTTGCTTGTGCACATGAGG

### Immunohistochemistry

Larvae were fixed in 4% PFA or 4% 1-ethyl-3 (3-dimethylaminopropyl)-carbodiimide overnight at 4℃. The larval brains were dissected in PBS, washed in PBS five times, and blocked with 4% goat serum, 0.1% Triton X-100, and 0.1% dimethyl sulfoxide in PBS overnight at 4 °C. Primary and secondary antibody staining was performed in respective blocking buffers overnight at 4 °C. Secondary antibodies were used at a dilution of 1:1000. The list of antibodies is rabbit anti-histamine 19C (1:10,000, [64, 66]), anti-tyrosine hydroxylase (Th1) monoclonal mouse antibody (1:1000, 22,941, Immunostar, Hudson, WI, [64]), HuC/D (16A11) mouse monoclonal IgG2b (1: 250, sc-56707, Santa Cruz, [67]), mouse monoclonal antibody to PCNA (1:1000, ab29, Abcam), and rabbit anti-gpr151 (1:5000, SAB4500418, Sigma,[17]). The following secondary antibodies were applied: Alexa Fluor 488, 568, or 647 goat anti-chicken, anti-mouse, or anti-rabbit IgG (1:1000, Invitrogen).

### Zebrafish Behavioral Assays

All larval and adult zebrafish tracking and analyses used the DanioVision system and Noldus Ethovision XT 13 program (Noldus, Wageningen, the Netherlands) for larval behavioral assay; after larvae were separated into 48-well plates, the plates were first habituated in the 28 °C incubator for 20 min. Both wild-type and homozygous larvae were analyzed on the same plate. All grouped adult fish were habituated in a 2.8-L plastic tank overnight for behavioral testing. All behaviors were analyzed between 9:00 and 17:00, except the sleep behavior, which was recorded for 24 h.

#### Larval Locomotor Activity and Thigmotaxis

Behavioral experiments were conducted in 48-well plates filled with 2 ml E3 medium. Six dpf larvae of each genotype were tracked for 15 min after 20-min acclimation. The thigmotaxis evaluation was performed by dividing each well into two zones, the periphery, and center of the well. Total distance moved, frequency, and cumulative duration in each zone were recorded using the DanioVision system and EthoVision XT software (Noldus Information Technology, Wageningen, The Netherlands).

#### Larval Dark-Flash Response

Zebrafish larvae have the innate tendency to increase their locomotion in response to a sudden change in the illumination of their environment. A burst of locomotor activity is normally observed during a period of sudden darkness, referred to as a “dark-flash” response [68]. Previous pharmacological evidence suggests that disrupted monoaminergic signaling leads to a stronger dark-flash response [52, 69]. Additionally, hrh1 antagonism has been reported to abolish the dark-flash response [8]. Thus, we decided to evaluate this behavior in zebrafish lacking hrh1. The dark-flash response of larvae was evaluated at 6 dpf as described before [52] with minor modifications. Briefly, after larval fish were habituated in the system for 20 min, the basic locomotor activity was recorded for 15 min. After that, larvae were exposed to alternating 2-min periods of darkness and light, with three periods of darkness in total. The index of dark response was calculated by the distances moved in the first 30 s of dark minus the last 30 s before dark, which is similar as described before [70]. Index (1st dark response) = (distances moved in 10 min-10 min 30 s) – (distances moves in 9 min 30 s – 10 min). The total distance moved was analyzed in 30-s bins. Larvae were individually tracked in 48-well plates using the DanioVision system and EthoVision XT software (Noldus Information Technology, Wageningen, The Netherlands).

#### Larval Acoustic/Vibrational Startle

To evaluate the startle response and sensory processing of zebrafish larvae, we utilized an acoustic/vibrational stimulus. This behavioral protocol has been described previously [25]. Briefly, larvae (6 dpf) were transferred from Petri dishes to 48-well plates filled with 1 ml E3 medium. The protocol (lights on) consisted of 30-min acclimation, followed by 10 acoustic/vibrational stimuli (DanioVision intensity setting 6) with a 20-s inter-stimulus interval (ISI), a 10-min pause followed by 30 vibrational stimuli with a 1 s ISI. Variable of interest to show the startle response was *maximum velocity* (mm/s) with 1-s intervals, since the startle response is a short burst of activity best captured by this parameter. Larvae were individually tracked using the DanioVision system and EthoVision XT software (Noldus Information Technology, Wageningen, The Netherlands).

#### 24h Locomotion Tracking

At 6 dpf, larvae of each genotype were tracked simultaneously for 24 h in 48-well plates, with the light conditions following the regular light/dark cycle of the larvae [71]. The trial started at 11:00 AM. The day and night activities were analyzed in 30 min bins by calculating the total distance moved. Larvae were individually tracked using the DanioVision system and EthoVision XT software (Noldus Information Technology, Wageningen, The Netherlands).

#### Adult Locomotor Activity and Thigmotaxis

Adult fish were individually tracked in separate cylindrical observation tanks (inner diameter 23 cm and water depth 5 cm), virtually divided into two zones (center and periphery) [72]. The fish had 5 min of habituation time in the tank before the tracking started. The swimming performance of the animals was automatically detected and tracked for 15 min by a digital video camera connected to a standard PC system running the EthoVision 13 software (Noldus Information Technology, Wageningen, The Netherlands). Total distance moved, frequency, and cumulative duration in each tank zone were the parameters evaluated.

#### Novel Tank Diving Test

Zebrafish have a natural tendency to spend more time at the bottom of the tank when placed in a novel environment before gradually migrating to the top. We used a novel tank diving assay to study anxiety-related risk-taking behavior [73]. Adult zebrafish were transferred from their home tank into the novel 1.5-L trapezoidal tank for behavioral observation and phenotyping. Novel tanks rested on a level, stable surface and were virtually divided into two equal portions (the bottom and upper part of the tank). Swimming behavior was recorded over a 6-min period without adaptation, and the time spent in each part of the tank was measured using EthoVision 13 software.

#### Shoaling Behavior

Zebrafish have the innate tendency to form shoals, a form of social interaction that serves many purposes in nature, including foraging, avoiding predation, and mating. Motivated by recent reports indicating a potential role for the histaminergic system in disorders where social behavior is impaired, we evaluated the shoaling behavior of WT and KO fish [52]. The protocol was carried out according to our previous report [60]. Five 6 mpf fish per cohort were placed in a round white polyethylene plastic flat-bottomed container (23-cm height, 23-cm diameter) with 2 L of fish system water (5.0-cm depth). Before testing, fish were habituated for 5 min followed by video recording for 10 min with a camera at a fixed height (60 cm) from the top of the container. All videos were analyzed with EthoVision XT 13 software using the default setting (the center-point detection of the unmarked animals). The average distance between subjects (defined as the distance between the body center of every member of the shoal) was quantified from the average data from all trials (4 trials, each trail contains 5 fish per genotype). The duration in proximity (in seconds) was defined as the cumulative duration of time a fish stayed close to the shoal fish (within 2 cm). The misdetection rate of the video-tracking software was less than 1%.

#### Statistical Analysis

Data was analyzed using GraphPad Prism version 7 software (San Diego, CA, USA). *p*-values were generated by one-way analysis of variance (ANOVA) for multiple comparisons using Tukey’s multiple comparison test, two-way ANOVA for multiple comparisons, and Student’s *t* test (unpaired test) for comparison of two groups. Data are presented as mean ± SEM. *p* value < 0.05 was considered statistically significant.

### Supplementary Information

Below is the link to the electronic supplementary material.

Supplementary file1 (PDF 511 KB)

## Data Availability

The datasets generated during and/or analyzed during the current study are available from the corresponding author on reasonable request.
