# Supplementary material for: The Roles of Histamine Receptor 1 (hrh1) in Neurotransmitter System Regulation, Behavior, and Neurogenesis in Zebrafish
Source: Mol Neurobiol. 2023 Jul 20;60(11):6660–75. doi: 10.1007/s12035-023-03447-z (PMC10533647; doi:10.1007/s12035-023-03447-z)
Supplement: Supplementary file 1 — Supplementary file1 (PDF 511 KB) [file 12035_2023_3447_MOESM1_ESM.pdf]

Supplementary figures 1-4 and legends

Supplementary fig. 1

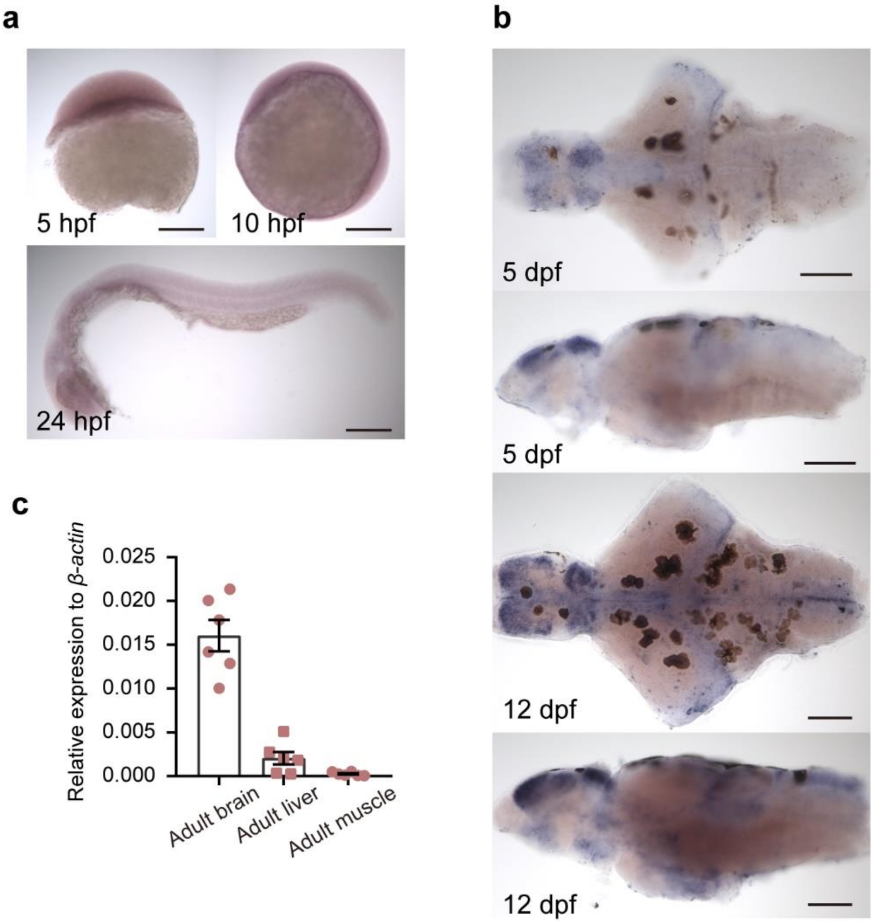

Supplementary figure 1: *hrh1* expression in development stage of Turku wt fish

a-b: *In situ* hybridization of zebrafish *hrh1* in Turku wt at 5 hpf, 10 hpf, 24 hpf, 5 dpf brain and 12 dpf brain. scale bar a: 100 μm, b: 50 μm. c: qPCR results of *hrh1* mRNA expression in adult brain, liver and muscle. N = 6. Mean ± SEM.

Supplementary fig. 2

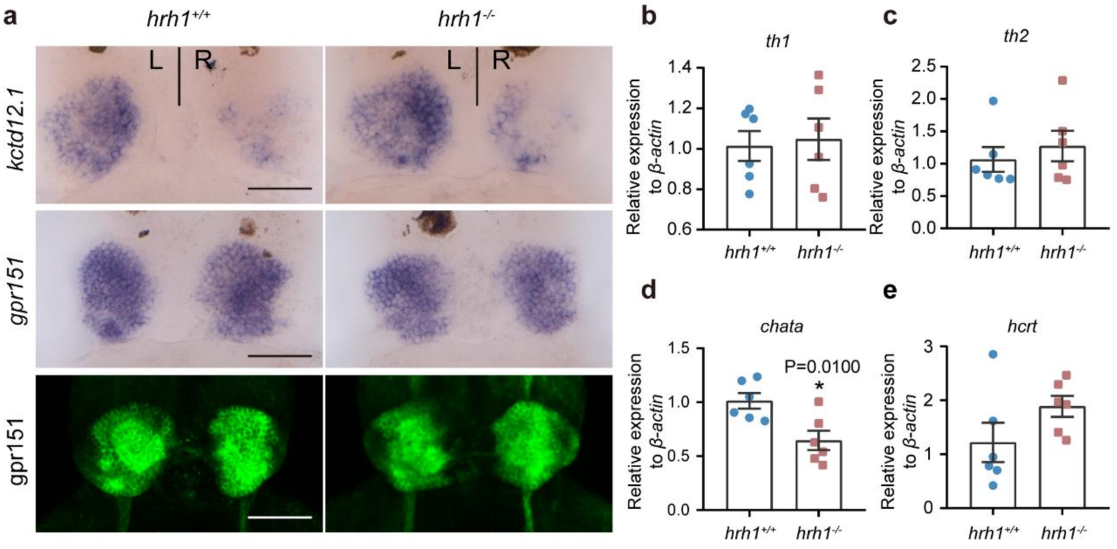

Supplementary figure 2: habenula markers in *hrh1*$^{-/-}$ larval brain and dopamine, *hcrt* expression in *hrh1*$^{-/-}$ adult brain

a: In situ and immunohistochemistry of *kctd12.1* and *gpr151* in habenula expression in *hrh1*$^{+/+}$ and *hrh1*$^{-/-}$ at 6dpf. N = 6. Scale bar: 50 µm. b-e: qPCR results of *th1*, *th2*, *chata*, *hcrt* in zebrafish adult brain. N = 6, Mean ± SEM, unpaired t test, two tailed. *: $0.01 < P \leq 0.05$; no stars mean no significant differences.

Supplementary fig. 3

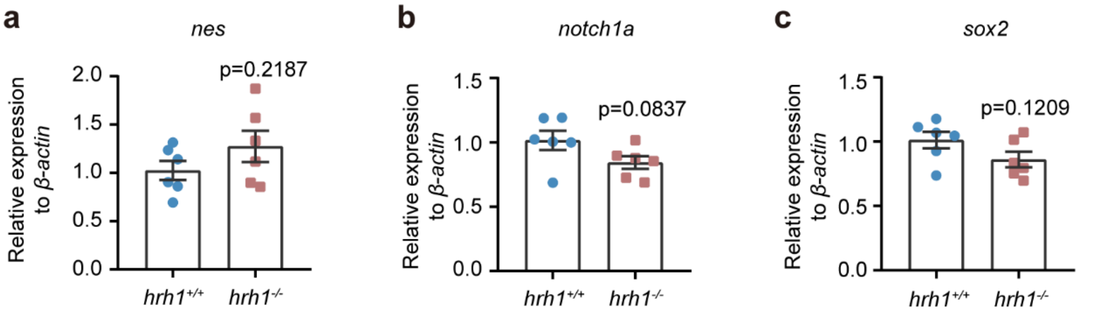

Supplementary figure 3: neurogenesis markers' change in adult *hrh1*$^{-/-}$ zebrafish brain

a-c: qPCR results of *nes*, *notch1a* and *sox2* in zebrafish adult brain. N = 6, Mean ± SEM, unpaired t test, two tailed. no stars mean no significant differences.

Supplementary fig. 4

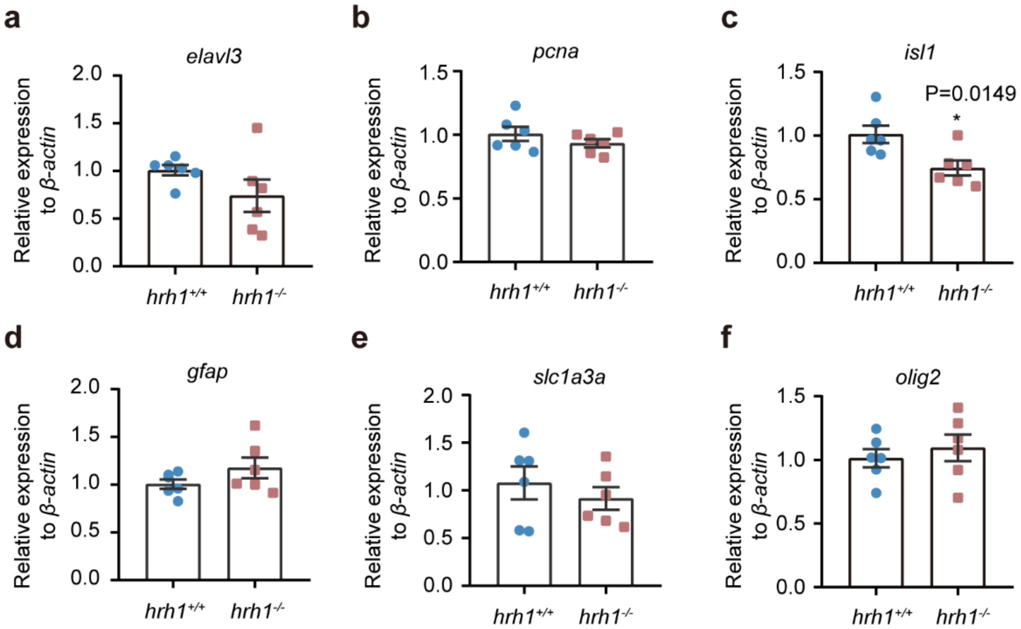

Supplementary figure 4: proliferation and differentiation markers' change in adult *hrh1*$^{-/-}$ zebrafish brain

a-f: qPCR results of *elval3*, *pcna*, *olig2*, *slc1a3a*, *gfap* and *isl1* in zebrafish adult brain. N = 6, Mean ± SEM, unpaired t test, two tailed. *: $0.01 < P \leq 0.05$; no stars mean no significant differences.
